# Approach to Low Body Temperature or Mild Hypothermia in the Geriatric Population: A Narrative Review

**DOI:** 10.14740/jocmr6490

**Published:** 2026-04-15

**Authors:** Prabhpaul Dhami, Kannayiram Alagiakrishnan

**Affiliations:** a Division of Internal Medicine, University of Alberta, Edmonton, Alberta, Canada; b Division of Geriatric Medicine, University of Alberta, Edmonton, Alberta, Canada

**Keywords:** Low body temperature, Mild hypothermia, Sepsis, Hypothyroidism, Polypharmacy, Chronic co-morbidities, Geriatric syndromes

## Abstract

While normal human body temperature is often cited as 36.1–37.2 °C, there is high-quality evidence demonstrating that older adults typically have lower baseline body temperatures. In contrast to hypothermia (< 35 °C) which is well recognized as a medical emergency, there is emerging evidence that low body temperature (35.0–36.0 °C) may predict poor outcomes in the geriatric population. This narrative review synthesizes the current literature on the association between low body temperature in adults aged ≥ 65 years and common geriatric co-morbidities. Across observational and cohort studies, low body temperature has consistently been associated with adverse outcomes in chronic kidney disease, hypertension, diabetes, chronic obstructive pulmonary disease, various malignancies, frailty, and neurodegenerative disorders. Despite these associations, in the absence of hypothermia, low body temperature remains a neglected topic in geriatric medicine. Recognition of low body temperature may improve early detection of geriatric co-morbidities, guide medication review, and identify patients at risk for cognitive decline and frailty. Further prospective studies are needed to clarify causal relationships and provide more insight into therapeutic implications.

## Introduction

The normal human core body temperature is often cited as 36.1–37.2 °C (97–99 °F), though estimates in the range of 35.5–37.5 °C have been cited as physiologically acceptable [1]. Hypothermia is defined as temperatures below 35 °C [2]. In adults older than age 60, a systematic review has found that measured body temperatures tend to be 0.23 °C lower on average compared to younger populations [3]. Low body temperature is defined as 35.0–36.0 °C. Though there is a reasonable amount of literature documenting the presence of low body temperature in older adults, it is frequently underrecognized in clinical practice. However, there is emerging evidence that suggests low body temperature may serve as diagnostic, therapeutic, or prognostic markers in a range of co-morbidities in the geriatric population [4, 5].

The elderly also make up a disproportionate share of the homeless population [6]. With social exclusion, lack of access to warm clothing or sustainable indoor shelter options, homeless patients have higher rates of hypothermic injury and mortality when compared to the general population [7]. When combined with the rising prevalence of mental health concerns amongst the elderly, including dementia or other intellectual disabilities, these patients may be unable to adequately judge weather conditions and risk exposure to potentially fatal weather extremes for prolonged periods of time [8].

In certain clinical scenarios, such as cardiac arrest, clinicians have historically employed a technique called therapeutic hypothermia, or targeted temperature management to improve neurologic outcomes [9]. However, recent large meta-analyses, such as the Targeted Hypothermia versus Targeted Normothermia after Out-of-Hospital Cardiac Arrest (TTM2) trial, have shown that hypothermia, with temperatures between 32 and 34 °C, provided no benefit for survivors of cardiac arrest when compared to strict normothermia with temperatures less than or equal to 37.5 °C [10]. In fact, the risk of adverse outcomes, such as infections, arrhythmias, and electrolyte disturbances was higher among patients with therapeutic hypothermia, which can significantly prolong recovery especially for geriatric populations [11].

Another common, but underrecognized cause of hypothermia-related deaths in old age is alcohol consumption [12]. Consuming alcohol impairs overall judgment, gives a false feeling of warmth in the presence of cold temperatures, and reduces the onset and duration of shivering [13]. Studies in rats have even shown that older age produces significantly greater reductions in body temperature after administration of ethanol [14]. Although the reason for these age-dependent differences remains unknown, alcohol metabolism is decreased in elderly people, making them more prone to its effects [15].

Although it is well known that elderly patients have a lower baseline body temperature, the clinical significance of low body temperature on geriatric health outcomes is an emerging topic that needs further research. In this narrative review, we have analyzed and presented the existing literature on low body temperature in the elderly in relation to geriatric co-morbidities.

### Search Strategy

A literature search was conducted using major electronic databases including MEDLINE, EMBASE, Scopus, and the Database of Abstracts of Reviews of Effects (DARE) from January 1965 to December 2025. The search strategy employed was designed to capture peer-reviewed studies relevant to the relationship between low body temperature and geriatric co-morbidities.

Search items were combined using Boolean operators. Temperature-related terms included “low body temperature” and “mild hypothermia.” These terms were paired with age-related terms including “older adults,” “geriatric patients” or “elderly.” Search items were also designed to capture geriatric co-morbidities and included “geriatric syndromes,” “cognitive impairment,” “dementia,” “Parkinson’s disease,” “polypharmacy,” “chronic obstructive pulmonary disease,” “frailty,” “chronic kidney disease,” “hypertension,” “diabetes,” “osteoporosis,” “decreased mobility,” “fractures,” “depression,” “anxiety,” and “falls.”

Studies were included if they discussed body temperature regulation, hypothermia, or low body temperature in adults greater than or equal to 65 years old, or if their findings were directly applicable to geriatric populations. Priority was given to original research articles, systematic reviews, meta-analyses, and large observational studies. Animal studies were also included selectively if they provided insight into thermoregulation or disease processes relevant to aging. Studies focused on pediatric populations, conference abstracts, or studies published prior to 1965 were excluded. As this was a narrative review, formal quality scoring was not performed. However, the included studies were critically appraised for clinical relevance, methodology, and applicability to geriatric populations.

## Physiology of Thermoregulation in the Elderly

The human body has developed several voluntary and involuntary mechanisms to remain normothermic, as normal functioning of organ systems and metabolic function at the molecular level are optimized at this temperature range [16]. Thermoregulation is a complex process that has been studied extensively in the literature. Centrally, body temperature is controlled by the hypothalamus which receives sensory input from thermal regulatory pathways throughout the body [17]. The anterior hypothalamus mediates heat dissipation by vasodilation and sweating, while the posterior hypothalamus is responsible for heat conservation by vasoconstriction and the shivering reflex. Core body temperature demonstrates remarkable homeostasis, even in the face of changes in geographic location or seasonable variations [18]. A large cross-sectional study measuring oral body temperatures found larger temperature ranges in patients living in locations of greater ambient temperatures and dew point variations, which may simply be due to known limitations of oral temperature probes [19]. Similarly, in otherwise healthy patients living in cold climates, core body temperature remains relatively constant due to physiologic responses such as increased brown adipose tissue thermogenesis [20]. As humans age, these voluntary and involuntary responses decline, heightening the risk of hypothermia in cold environments [16].

**Figure 1:**
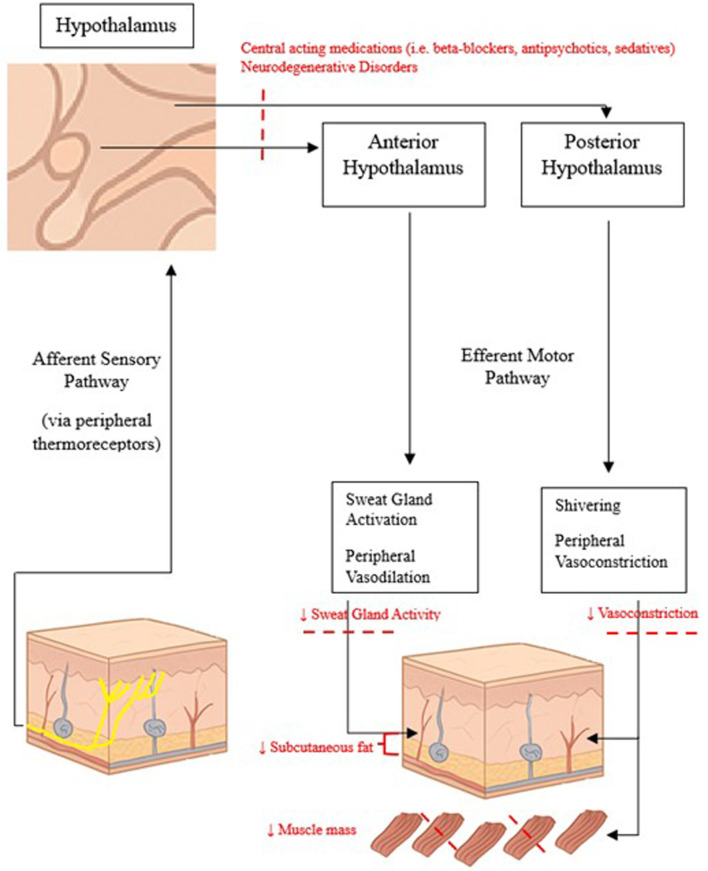
Normal thermoregulatory pathways and changes associated with aging.

There are several postulated mechanisms that contribute to the blunted thermoregulatory response in older adults (Fig. 1). Age-related decrease in subcutaneous fat, reduced metabolic rate, impaired peripheral vasoconstriction, and decreased sweat gland function all contribute to the body’s inability to maintain core body temperature when exposed to cold temperatures [16, 21]. Additionally, co-morbidities such as hypothyroidism, anemia, and neurodegenerative disorders can exacerbate susceptibility to low body temperature [22]. Age-related reduction in cardiovascular and musculoskeletal function, including lower physical activity, can compromise metabolic heat production needed to maintain normal body temperatures [23, 24]. These effects are demonstrated even when older patients are performing normal activities of daily living [25]. Additionally, sex hormones may also contribute to age-related changes in thermal regulation [26]. Progesterone levels, which promote heat conservation through peripheral vasoconstriction and centrally through changes in thermoregulatory set-points, drop drastically in post-menopausal females. The effect of androgens in male patients is more complex, but the literature consistently demonstrates that lower testosterone levels in aging males leads to decreased basal metabolic rate and muscle stores, which may contribute to lower body temperatures [27, 28]. Medications commonly used by the elderly population, including beta-blockers, antipsychotics, and sedatives, can also predispose patients to lower body temperatures [29]. As climate change is causing larger temperature extremes, targeting the aging population with reduced thermoregulatory capacity becomes even more crucial [30].

## Challenges With Body Temperature Measurement

There are many sites where body temperature can be measured (Table 1). Most commonly in practice, practitioners use ear (tympanic membrane), oral, rectal, axillary, temporal artery (forehead), and nasopharyngeal temperature probes to measure body temperature [31]. There are more invasive probes, such as pulmonary artery, esophageal, or urinary bladder measurements that are often restricted to inpatient or critical care settings [32, 33]. Each modality of temperature measurement has its own unique advantages and disadvantages. Sites that are close to highly perfused organs or great vessels are deemed to be the most reliable in measuring core temperatures [34]. The classic example of this is the Swan-Ganz catheter, which measures temperature directly from the pulmonary artery [35]. These are known to provide precise and repeatable measurements but are invasive and only practical in intensive care units. The long esophageal thermometer measures temperature in the distal esophagus, which is near the left atrium and ventricle, making it strongly associated with pulmonary artery temperature [36]. Additionally, esophageal temperature changes fairly quickly in changes to core temperature, and does not require intravascular access, making it the preferred measurement technique in intubated patients during surgery [34]. Of note, during rapid cooling and rewarming, as seen in cardiac surgery, esophageal temperatures have been observed to lag behind vascular probes [37]. Another less invasive option for temperature measurement includes nasopharyngeal temperature probes [38]. These probes are commonly used to monitor temperatures during general anesthesia due to its anatomical proximity to the internal carotid artery [39]. However, these thermometers are inherently difficult to position [40]. Previous studies have shown that trained residents and nurses only correctly place the probe in the upper or mid-nasopharynx 41% to 43% of the time. A more reproducible and precise measurement option includes urinary bladder catheters with temperature sensing [32]. This method is precise and easy to use for inpatients with indwelling urinary catheters. These perform well for patients with steady thermal states, but do not perform well with rapid changes in temperature as seen in cardiopulmonary bypass [32]. Oral and axillary temperatures, while convenient, are often plagued by inconsistencies as they are affected by thermometer placement, ambient temperatures, ingestion of food, and smoking [19]. Oral temperatures do not accurately reflect core body temperature, and in emergency situations, such as exertional heat stroke, temperature readings will grossly underestimate and delay diagnosis and treatment [41]. For this reason, oral and axillary temperatures are adjusted by adding 0.3 and 0.5 °C, respectively [42]. Tympanic and temporal artery thermometers employ an infrared radiation device to measure temperature, which are faster and convenient to use [43]. Widespread use of these infrared sensors, especially non-contact forms such as the forehead thermometer, has become commonplace after the coronavirus pandemic [44]. These non-contact thermometers have their own unique advantages, including decreased patient distress from sleep interruption which is often necessary for other forms of measurement [45]. Especially for elderly patients with sleep-wake disturbances from dementia, these disruptions can be particularly bothersome. Tympanic membrane thermometry is another non-intrusive and accurate method of measuring temperature in older adults, and is likely the most widely used in clinical practice [46]. There is also a theoretical advantage with the ear site, as the tympanic membrane and hypothalamus share a common blood supply [31]. A study has also shown that tympanic thermometers offer a close approximation of pulmonary artery temperature, making them practical for outpatient and home use [47]. Under appropriate conditions, rectal temperature probes demonstrate good agreement with intravascular central thermometers [48]. While once widely used due to its convenience and proximity to large pelvic arteries, more recent research has shown large discrepancies in temperature measurement, mostly attributed to poor placement technique and local conditions such as rectal inflammation and presence of feces [49]. Additionally, as with many temperature measurement techniques, there is a delayed response to changes in rectal temperature during rapid core temperature changes [50]. There are also many examples of enteric pathogens spreading via incorrect usage of rectal temperature probes [51]. With the right precautions, many of the risks and downsides with rectal measurement, such as patient embarrassment or risk of nosocomial infection, can be reduced [31].

**Table 1:** Comparison of Body Temperature Measurement Methods in Elderly Patients

Accuracy rank	Measurement site	Instrument/technique	Advantages	Disadvantages	References
Best	Pulmonary artery	Swan-Ganz catheter	1. Gold standard for measuring core body temperature as measures directly from central circulation. 2. Highly precise and repeatable.	1. Highly invasive, requires intravascular access. 2. Limited to intensive care unit (ICU) and operative settings.	Hymczak et al, 2021 [35]
	Distal esophagus	Long esophageal thermistor	1. Strongly correlated with pulmonary artery temperature. 2. Changes rapidly in response to core temperature changes. 3. Preferred in intubated patients.	1. Lags during rapid rewarming/cooling as seen in cardiac surgery. 2. Not useful for awake patients.	Lefrant et al, 2003 [36]; Misra et al, 2023 [37]; Sessler, 2016 [34]
Good	Nasopharynx	Nasopharyngeal temperature probe	1. Anatomically close to internal carotid artery. 2. Reflects core temperature during anesthesia. 3. Widely available.	1. Difficult to position correctly (highly practitioner dependent).	Lee et al, 2014 [40]; Lim et al, 2016 [39]
	Urinary bladder	Temperature-sensing urinary catheter	1. Offers continuous temperature monitoring. 2. Reliable and precise during steady states.	1. Temperatures lag behind rapid core temperature shifts (as seen in cardiopulmonary bypass). 2. Limited to patients with urinary catheters.	Fallis, 2002 [32]
	Rectum	Rectal probe (≥ 15 cm insertion)	1. Easy to use. 2. Correlates well with intravascular temperatures under stable conditions.	1. Delayed response during rapid temperature change. 2. Temperatures are affected by presence of feces or inflammation. 3. Risk of transmitting infection (e.g., VRE transmission). 4. Patient discomfort.	Robinson et al, 1998 [50]; Livornese et al, 1992 [51]; Niven et al, 2015 [48]
Moderate	Tympanic membrane	Infrared or insulated thermistor	1. Non-invasive. 2. Approximates brain core temperature (shares blood supply with hypothalamus).	1. Accuracy reduced by cerumen, ear pathology, or poor placement technique.	Mah et al, 2021 [47]; Gasim et al, 2013 [46]; Sund-Levander & Grodzinsky, 2020 [31]
Poor	Temporal artery (forehead)	Infrared temporal scanner	1. Contactless and thus low infection transmission risk. 2. Does not require patient to be awake.	1. Highly influenced by ambient temperature, sweat, and poor technique. 2. Does not correlate well with true core temperature.	Chen et al, 2020 [43]; Hussain et al, 2021 [44]; Chen et al, 2021 [45]
	Oral	Oral electronic thermometer	1. Convenient, accessible. 2. Non-invasive and widely available.	1. Inaccurate during thermal stress, recent oral intake, or after smoking. 2. Underestimates true core body temperature and possible fever.	Mazerolle et al, 2011 [41]; Betta et al, 1997 [42]; Ley et al, 2023 [19]
	Axilla	Axillary thermometer	1. Widely available and easy to use. 2. Safe alternative when oral or rectal routes are contraindicated.	1. Poorly correlates with core body temperature. 2. Strongly affected by ambient temperature and skin moisture.	Sund-Levander & Grozsinsky, 2020 [31]; Ley et al, 2023 [19]

Elderly patients present unique challenges in the measurement of body temperature. The frail elderly often present with infection atypically, with core body temperature in the normal range [52]. Due to the lack of the cardinal sign of fever, traditional body temperature measurement techniques may delay diagnoses in elderly patients, thus leading to worse outcomes. Additionally, antipyretics, such as acetaminophen, are commonly used in this age range and may artificially blunt the febrile response [53]. Peripheral (temporal artery, axillary, oral, or tympanic membrane) thermometers have a low sensitivity for detecting the low-grade fever seen in elderly patients [48]. Even though rectal thermometers remain the gold standard in assessing temperature in geriatric populations, there are some downsides to consider. For example, rectal temperatures may be affected by impacted stool from constipation, and rectal incontinence, both of which are conditions that affect the elderly at a higher prevalence than the general population [54, 55]. In the event that a rectal temperature cannot be taken, or is suspected to be unreliable, peripheral thermometers may be used, but care must be taken to follow manufacturer guidelines on technique and placement [56].

## Possible Temperature Ranges in the Elderly

With these variations in body temperature in older adults, along with existing research on normal temperature ranges, we propose a revised range of normal body temperatures for the geriatric population (Table 2).

**Table 2:** Revised Body Temperature Range for Adults ≥ 65 Years Old

Clinical correlate	Temperature range (°C)
Hypothermia	< 35.0
Low body temperature	35.0–36.0
Normothermia	36.1–37.2
Low-grade fever	37.3–37.9
True fever	≥ 38

The definition of a low-grade fever is a hotly contested topic in the literature, with several studies showing that lower temperature cutoffs improve sensitivity and infection detection rates in the elderly population [57]. American clinical practice guidelines have suggested repeat oral temperatures > 37.2 °C, especially in older adults with lower baseline body temperatures, may represent a more appropriate lower limit for low-grade fever. One systematic review on COVID-19 patients found that patients were significantly more likely to have a low-grade fever, defined as 37.2–38.0 °C, compared to a high-grade fever of 39.0 °C. In healthy community-dwelling elderly patients, studies have documented the lower limit of normal body temperature equal to 36.1 °C [58, 59]. Notably, with the consensus definition of hypothermia being less than 35.0 °C, this leaves the low body temperature range of 35.0–36.0 °C, which has not been well studied in the literature [2].

In the absence of a traditional fever, or a body temperature of greater than or equal to 38 °C, clinicians may mistakenly overlook the early signs of infection or malignancy [19]. Of course, clinical context is always paramount, and localizing signs of infection are necessary before prescribing broad-spectrum antibiotics which may have their own adverse side effects [60].

## Low Body Temperature and Common Co-Morbidities in the Elderly

### Kidney disease

Acute and chronic kidney disease (CKD), among other chronic medical conditions and geriatric co-morbidities, has been associated with low body temperature and hypothermia (Table 3). In moderate hypothermia, glomerular filtration rate declines with falling cardiac output, leading to “pre-renal” kidney failure [61]. In practice, over 40% of patients admitted to the intensive care unit for accidental hypothermia experience acute kidney injury. Although there is a lack of systematic data regarding the correlation between hypothermia and kidney disease, there are case reports describing hypothermia-induced kidney injury occurring in elderly patients [62]. Due to a lack of hypothermia awareness in elderly patients, these repeated renal insults may progress to CKD. One study found that 46% of patients with accidental hypothermia had some form of renal damage, 17% of which were exacerbations of CKD [63]. Another study analyzed 14 elderly patients admitted to hospital due to hypothermia in the winter and found elevated serum creatinine in eight of them. Especially in northern climates, where winter can exceed 6 months, these cases of accidental hypothermia become more frequent [64]. Combined with other co-morbidities in elderly patients that are known to be deleterious to kidney health, such as diabetes mellitus, these hypothermia events may accelerate progression of CKD.

**Table 3:** Relationship Between Low Body Temperature and Geriatric Co-Morbidities

Comorbidity	Pathophysiologic link to low body temperature	Clinical consequences	Supporting references
Chronic kidney disease (CKD)	1. Hypothermia causes decreased cardiac output and lowers glomerular filtration rate leading to pre-renal injury. 2. Endogenous cryogens and uremic toxins suppress normal thermoregulatory responses. 3. CKD-related malnutrition and anemia lead to impaired thermogenesis and reduced heat conservation.	1. High prevalence of acute kidney injury (AKI) in hypothermic patients. 2. Hemodialysis patients have a lower baseline body temperature prior to dialysis. 3. Cold skin and cold intolerance in CKD-associated anemic patients, improving with erythropoietin administration.	Mallet, 2002 [61]; Kuriyama et al, 1999 [63]; Yamada et al, 2010 [62]; Noe et al, 2012 [64]; Kluger et al, 1981 [65]; Ash, 1991 [66]; Fishbane & Spinowitz, 2018 [67]; Brigham & Beard, 1996 [68]; Ludwig & Strasser, 2001 [69]; Grassi et al, 2003 [21]; Kenney & Munce, 2003 [16]
Hypertension	1. Cold exposure leads to increased sympathetic nervous system activity and RAAS activation, which causes vasoconstriction and increased vascular resistance.	1. Cold-induced blood pressure elevation. 2. Higher mortality in hypertensive elderly during winter months.	Goel et al, 2022 [70]; Hu et al, 2021 [71]; Wang et al, 2017 [72]; Greaney et al, 2017 [73]; Qi et al, 2024 [74]
Diabetes mellitus	1. Autonomic and small fiber neuropathy leads to impaired shivering, vasoconstriction, and thermoregulation. 2. Hypoglycemia may precipitate or result from hypothermia.	1. Increased cold-related morbidity and mortality. 2. Frequent hypoglycemic episodes in elderly patients during low ambient temperatures.	Cheshire, 2016 [77]; Tran et al, 2012 [78]; Lai et al, 2020 [79]; Song et al., 2021 [76]; Yang et al, 2016 [75]; Ali Imuran et al, 2018 [80]; American Diabetes Association Professional Practice Committee, 2025 [81]
Chronic obstructive pulmonary disease (COPD)	1. Chronic hypoxemia and polyneuropathy impair autonomic thermoregulation. 2. Cold exposure worsens pulmonary function.	1. Increased exacerbations and hospitalizations during cold weather. 2. Low body temperature correlates with disease severity.	Yang et al, 2024 [82]; Lam et al, 2018 [83]; Li et al, 2022 [84]; Aras et al, 2018 [86]; Chhabra & De, 2005 [87]; Arisoy et al, 2021 [85]
Malignancy	1. Cold-induced sympathetic activation and β-adrenergic signaling promote carcinogenic pathways, angiogenesis, and metastasis formation. 2. Sarcopenia from malignancy leads to decreased insulation and heat retention.	1. Increased cancer incidence, mortality, and treatment resistance in colder climates. 2. Increased susceptibility to hypothermia in sarcopenic patients.	Bandyopadhayaya et al, 2020 [88]; Mravec & Tibensky, 2020 [90]; Eng et al, 2015 [91]; Zhang et al, 2023 [92]; Obermeyer et al, 2017 [93]; Sørensen et al, 2005 [94]
Parkinson’s disease	1. Central thermoregulatory dysfunction from α-synuclein deposits and Lewy bodies in hypothalamus. 2. Peripheral autonomic and sensory denervation.	1. Impaired sweating, vasodilation, and shivering via normal autonomic regulatory pathways. 2. D2 receptor agonist mediated thermoregulatory changes.	Coon & Low, 2018 [95]; De Marinis et al, 1991 [97]; Doppler et al, 2014 [96]; Hama et al, 2009 [98]
Polypharmacy	1. Impaired thermoregulatory mechanisms via anxiolytics, antipsychotics, antidepressants, and opioids. 2. Weakening compensatory response to low ambient temperatures (β-blockers, α1-adrenergic receptor agonists, and oral antihyperglycemics).	1. Drug-induced hypothermia. 2. Impaired recognition of hypoglycemia. 2. Altered drug metabolism in mild hypothermia.	Van Marum et al, 2007 [29]; Zonnenberg et al, 2017 [101]; Nemmani et al, 2001 [104]; Liu et al, 2016 [102]; Vue & Setter, 2011 [110]; Lin et al, 1984 [112]; Tortorici et al, 2007 [113]; Ben-Uriah et al, 1981 [109]
Dementia/cognitive decline	1. Low body temperature leads to tau hyperphosphorylation and amyloid accumulation. 2. Hypothalamic involvement in neurodegeneration.	1. Worsening cognition. 2. Increased risk of conversion to dementia. 3. Low body temperature as a prodromal phase of dementia.	Vandal et al, 2016 [116]; Blessing et al, 2022 [117]; Van De Nes et al, 1998 [118]; Alagiakrishnan et al, 2023 [119]; Yamagiwa et al, 2025 [115]; Fischer et al, 2024 [120]; Wang et al, 2025 [121]
Frailty	1. Low body temperature is associated with reduced metabolic rate, loss of muscle mass, and impaired physiologic reserve, all of which contribute to frailty.	1. Lower body temperatures are associated with increased risk of frailty. 2. Frail patients living in colder temperatures have more rapid progression in frailty.	Hoogendijk et al, 2019 [122]; Chen et al, 2014 [123]; Alakare et al, 2022 [124]; Cesari et al, 2017 [125]; Takauji et al, 2021 [126]; Zhou et al, 2023 [127]; He et al, 2025 [128]

Low body temperature has also been discussed in relation to CKD in the literature. A study in rabbits found that injection of human urine resulted in a fall in body temperature, along with peripheral vasodilation and suppression of the normal shivering reflex in response to cold temperatures [65]. In addition, oral temperatures on end-stage renal disease patients were noted to be lower before hemodialysis runs, suggesting the presence of an endogenous dialyzable cryogen responsible for these temperature changes. In patients with severe uremia, with blood urea nitrogen rising near 100 mg/dL, uremic hypothermia has also been observed [66]. The mechanism by which uremic toxins lead to a drop in body temperature is unknown but thought to be related to the disruption of normal gradients across cell membranes, direct toxin effects of waste products on cells, as well as changes in glucose metabolism. Moreover, renal dysfunction is well known to cause anemia secondary to a relative deficit of erythropoietin and iron deficiency [67]. Patients with iron deficiency anemia have poor temperature regulation capacity, likely due to effects on both heat production and heat loss rates [68]. Moreover, patients with anemia frequently report cold skin as a bothersome symptom that may actually improve with administration of recombinant human erythropoietin [69]. Patients with CKD also frequently suffer from malnutrition, which is well known to cause low body temperature due to reduced insulating subcutaneous fat and decreased skeletal muscle mass for heat production [16, 21].

### Hypertension

There is a robust inverse relationship between low body temperature and blood pressure, whereby epidemiologic studies have demonstrated increases in both systolic and diastolic blood pressure in the face of cold environments [70]. Cold exposure drives activation of the sympathetic nervous system and the renin-angiotensin-aldosterone axis, leading to vasoconstriction and increased peripheral vascular resistance. This relationship applies to patients of all populations, but is particularly significant in older adults with pre-existing hypertension [71]. A systematic review and meta-analysis has demonstrated that even a 1 °C decrease in ambient temperature is associated with an increase in systolic and diastolic blood pressure by 0.26 and 0.13 mm Hg, respectively [72]. This effect was also demonstrated in a whole body cooling study whereby hypertensive patients experienced greater increases in mean arterial pressure when reducing their skin temperature from 34.0 to 30.5 °C [73]. Low body temperature- and hypothermia-related blood pressure elevations likely contribute to morbidity and mortality, as studies have demonstrated higher rates of non-accidental death among hypertensive elderly patients during the winter months [74]. Despite high-quality evidence demonstrating that low environmental and body temperatures are associated with poorer blood pressure control in geriatric patients, temperature is rarely considered in hypertension management [70].

### Diabetes

Low body temperature and hypothermia are linked to increased morbidity and mortality in patients living with diabetes [75, 76]. One multi-city series study in China estimated that mortality related to cold exposure was particularly high amongst elderly patients with diabetes. The mechanism for this relationship is multifaceted, but is postulated to be the effect of small fiber and autonomic neuropathy secondary to poorly controlled diabetes [77]. Involuntary responses by the sympathetic nervous system, including vasoconstriction and shivering, are impaired by autonomic neuropathy, which is present in approximately 10% of patients with diabetes. Furthermore, hypothermia has been noted to be a common manifestation of severe hypoglycemia [78]. One study found hypothermia in about a quarter of patients with severe hypoglycemia that presented to the emergency department.

Interestingly, this association seems to work in reverse as well, as an ecological study in Taiwan found that low ambient temperatures were correlated with a significant increase in hypoglycemic episodes [79]. This same study found that the incidence rate of hypoglycemia was significantly higher in those aged greater than or equal to 65 years old. Many contemporary practice guidelines for diabetes management have recognized the increased risk for hypoglycemia in the elderly, and have adopted more lenient hemoglobin A1C targets as a result [80, 81]. In geriatric patients, using antihyperglycemics with lower hypoglycemia risk and routine fasting blood glucose level monitoring should be integrated into practice, particularly for those with low body temperature and during colder months.

### Chronic obstructive pulmonary disease

As with diabetes, patients with chronic obstructive pulmonary disease (COPD) tend to have poor outcomes in periods of lower ambient temperatures [82]. Acute exacerbations of COPD occur more frequently in winter months, with the highest risk observed among patients aged ≥ 65 years. Moreover, high temperature seasons (> 28 °C) have also been correlated with increased pneumonia hospitalizations in elderly patients with COPD [83]. The literature on this topic is primarily focused on environmental temperature; however, one study has found that low measured body temperature may be an indicator of disease severity in stable COPD patients relative to healthy controls [84]. Specifically, lower body temperatures were found to be correlated with a higher smoking index and increased dyspnea as defined by the modified Medical Research Council (mMRC) dyspnea scale. The postulated mechanism by which low body temperature correlates with poor lung function may be due to polyneuropathy, a well described feature of advanced COPD [85]. Chronic hypoxemia impairs nerve oxygenation, which may lead to both peripheral and autonomic neuropathy [86, 87]. As with other causes of neuropathy, this impairs the normal homeostatic mechanisms to maintain normal core body temperatures, especially through poor peripheral circulation. Overall, it appears that exposure to temperature extremes places elderly patients with COPD at higher risk of hospitalization for respiratory illnesses, and that low baseline body temperatures may serve as a marker of disease severity in this population.

### Malignancy

Epidemiological studies have also found that patients living in colder regions have higher incidence and mortality rates for various malignancies [88]. Sub-group analyses have shown that women over 65 years of age tend to be at the highest risk. Studies in the United States and global datasets show the strongest association between low environmental temperatures and breast, melanoma, leukemia, pancreatic, bladder, uterine, thyroid, non-Hodgkin’s lymphoma, esophageal, ovarian, brain, and gastroesophageal cancers [89]. Animal studies investigating the correlation between cold temperatures and increased incidence of cancer suggest cold-induced sympathetic activation and norepinephrine release may be the driver of carcinogenesis [90]. These studies have shown that prolonged beta-adrenergic receptor activation can activate oncogenes, induce DNA mutations, prevent malignant cell apoptosis, and even promote angiogenesis and development of metastases. One study conducted on mice demonstrated that tumor susceptibility to cisplatin was significantly enhanced when raising ambient temperatures from 22 to 30 °C [91]. Additionally, patients with cancer are more likely to be sarcopenic due to malignancy-related catabolism and chemotherapy-induced muscle loss, which increases their risk of hypothermia [92]. Moreover, the link between low resting body temperature and malignancy is less clear. In fact, in large data analyses, resting body temperature tends to be slightly elevated in patients with active malignancy [93]. Hematologic malignancies, especially lymphoma, leukemia, and multiple myeloma are often associated with drenching night sweats and fevers [94]. Overall, cancer incidence, mortality, and treatment resistance is higher in patients residing in cold climates; however, the relationship between prodromal resting body temperatures and malignancy has not been studied.

### Parkinson’s disease

Another condition that may be associated with low temperature and hypothermia is Parkinson’s disease. Many manifestations of Parkinson’s disease may be responsible for the thermoregulatory dysfunction noted in these patients [95]. Broadly speaking, Parkinson’s disease affects both central and peripheral mechanisms of thermoregulatory control. Deposits of alpha-synuclein and Lewy body formation in regions of the brain, such as the hypothalamus, may be a cause for central deficits in thermoregulation. Peripheral neurodegeneration in these patients has also been described in patients with Parkinson’s. Loss of peripheral nerve fibers may affect cutaneous responses to ambient temperature fluctuations, such as sweating, erector pili muscle function, and vasodilation [96]. Indeed, one study found that patients suffering from Parkinson’s disease had reduced sweating and superficial vasodilation compared to control subjects, especially on the symptomatic side of their body [97]. Parkinson’s patients have also been noted to lack the shivering response to create heat when faced with hypothermia [95]. Important confounders in studies linking Parkinson’s disease to low temperature are the medications these patients take. Drugs administered for the treatment of Parkinson’s disease, such as dopamine receptor agonists, may affect thermoregulatory mechanisms as well [98].

## Low Body Temperature and Geriatric Syndromes

### Polypharmacy

A common cause for low temperature abnormalities or hypothermia in elderly patients is medication use. Given the fact that polypharmacy is a common issue in the elderly, most of the medications discussed are taken at a higher proportion in geriatric patients relative to the general population [99].

One mechanism by which medications may contribute to hypothermia is through disruption of normal thermoregulatory processes [100]. Drug classes commonly implicated in this mechanism include anxiolytics, antipsychotics, antidepressants, and opioids. Hypothermia is a rare, although well documented side effect of antipsychotic medications [29]. Antipsychotics that strongly antagonize 5-HT2 receptors seem to cause hypothermia more often [101]. Opioid medications affect body temperature through binding to µ-, δ-, or κ-opioid receptors [102]. Studies in mice have shown that administration of opioids that agonize µ- and κ-opioid receptors results in mild hyperthermia and hypothermia, respectively. Hypothermia-related deaths have also been associated with antidepressant use, although the exact mechanism is currently unclear in the literature [103]. Research in rats has demonstrated that fluoxetine, a selective 5-HT reuptake inhibitor, may potentiate body temperature reductions [104]. In effect, geriatric patients who take these combinations of medications may be more likely to experience a drop in body temperature and hypothermia.

Another way medications may reduce body temperature is by weakening homeostatic responses to cold exposure [105–107]. Drugs that fall under this category include oral antihyperglycemics, beta-blockers, and alpha 2-adrenergic agonists. Oral antihyperglycemics are more frequent causes of accidental hypoglycemia, which is one of the causes of hypothermia [108]. Lowering metabolic rate, and thus lowering body temperature, may be a protective mechanism when the body faces inadequate fuel supply. The exact mechanism behind beta-blocker-induced hypothermia has been discussed in the literature but remains unclear [109]. It has been postulated that beta-blockers act in the central nervous system (CNS) on temperature regulation pathways to cause lowering of body temperature. Additionally, beta-blockers can induce or potentiate hypoglycemia [110]. As beta-blockers are known to mask catecholamine-mediated hypoglycemic symptoms, such as tremor and hunger, these agents may delay recognition and treatment of hypothermia from these medications. Clonidine, an alpha 2-adrenergic agonist, has been studied for its effects on thermoregulatory responses in multiple species [111, 112]. Studies in cats and rats have shown clonidine causes a reduction in core body temperature, which may be linked to alpha-adrenergic receptors found in the anterior hypothalamus.

Moreover, mild hypothermia alters pharmacokinetics of several medications, especially those metabolized in the liver [113]. Systemic clearance of drugs by cytochrome P450 has been cited to decrease by 7–22% per degree Celsius below normal core body temperature. Changes to hepatic metabolism may account for morbidity and mortality amongst elderly patients with low body temperature, especially for those on agents with narrow therapeutic indexes [114].

### Dementia

An emerging topic in the literature is the association between low body temperature and cognitive decline in the elderly [115]. Recent cross-sectional studies have found that relative hypothermia is independently correlated with worsening cognitive function and increased gray matter atrophy using magnetic resonance imaging in patients > 60 years old. The pathophysiology of temperature associated cognitive decline has been explored in animal models, and one study has demonstrated that transgenic mice with low temperatures have increased abnormal tau proteins, which is a pathologic marker of Alzheimer’s dementia [116]. This same study also demonstrated that elevation of environmental temperatures resulted in decreased brain plaques and normalization in memory deficits in mice relative to controls. A clinical trial in elderly human patients also showed that tau hyperphosphorylation, an indicator of neurodegenerative disease, significantly increased in cerebrospinal fluid (CSF) and plasma fluid samples with small decreases (< 1 °C) in body temperature [117]. Previous research has also demonstrated that amyloid plaques, another hallmark of Alzheimer’s disease, have been found in the hypothalamus, the primary brain region responsible for body temperature regulation [118]. Additionally, in patients with pre-existing mild cognitive impairment, a retrospective study has demonstrated that low body temperature is independently associated with conversion to dementia [119]. In this sense, low body temperature may represent a prodromal stage of dementia. As body temperature begins to fall below 35 °C into more severely hypothermic states, a step-wise decrease in cognition occurs to the point of patients reaching unconsciousness below 28 °C [120]. Although instances of severe hypothermia can induce protein denaturation and apoptosis leading to neuronal death, even milder forms of low body temperature over time may pre-dispose patients to a major neurodegenerative disorder later in life [121]. Elderly patients with documented low body temperature need to be screened early for cognitive decline.

### Frailty

Frailty is a multifaceted condition characterized by a decline in multiple organ systems, leaving patients more vulnerable to external stressors [122]. Many of the pathologic mechanisms associated with low body temperature, such as diminished metabolic rate, lower muscle mass, and geriatric syndromes, also put patients at risk for frailty [123]. One study examining body temperature measurements in community-dwelling elderly residents found that patients with lower tympanic and rectal temperatures were more likely to have impaired activities of daily living, dementia, and body mass index, all of which are key components of commonly used frailty measures.

Furthermore, the association between low body temperature and frailty also has clinical implications in acute care settings [124]. A large-scale study found that frail geriatric patients with low body temperature who were evaluated in the emergency department had significantly higher 30- and 90-day mortality [124]. Given their limited physiologic reserve, patients with documented frailty poorly tolerate external stressors, including temperature extremes [125]. One study reported that patients who presented to care with accidental hypothermia had a higher mortality if they were classified as frail according to the Clinical Frailty Scale [126].

Environmental temperature may also influence frailty risk. A Chinese longitudinal study found that patients living in lower ambient temperatures had high frailty indices compared to those living in warmer climates [127]. This relationship has also been demonstrated longitudinally, with a recent study showing older adults living in colder environments were more likely to experience rapid progression in frailty [128]. Frailty remains an evolving area of geriatric medicine that provides clinicians with an overarching understanding of a patient’s clinical status [122]. Although the literature on this topic is sparse and in need of further studies, the presence of low body temperature may serve as a potential surrogate marker of frailty.

## Practical Interventions for Managing Low Body Temperature

Given that low body temperature may be correlated with poor outcomes in the elderly, and that some animal models even show that correcting low body temperatures can reverse this effect, clinicians need a structured approach to evaluating and treating low body temperature (Table 4). Firstly, all geriatric patients should be screened for low body temperature in routine evaluations. Although rectal temperatures may be more reflective of core body temperatures, they are often not practical and uncomfortable for geriatric patients [31, 43]. We suggest using tympanic thermometers as they are easy to use, and offer a theoretical advantage being close to the hypothalamus, which is the thermoregulatory center in the brain.

**Table 4:** Practical Interventions for Low Body Temperature in Older Adults

Area of focus	Practical interventions and management strategies	Clinical rationale	Supporting references
Screening and measurement	1. Measure temperature in all clinical assessments of geriatric patients. 2. Use tympanic thermometers for practicality.	Tympanic readings provide a reasonable estimate of core temperature in the elderly and have close proximity to the thermoregulatory center of the brain.	Chen et al, 2020 [43]; Sund-Levander & Grodzinsky, 2020 [31]
Nutritional and musculoskeletal health	1. Ensure adequate caloric and protein intake. 2. Encourage resistance training to maintain muscle mass. 3. Refer to dietitian or physiotherapist for individualized plans.	Malnutrition and sarcopenia lower basal metabolic rate, reduce subcutaneous insulation, and predispose patients to hypothermia and strength-related functional decline.	Grassi et al, 2003 [21]; Kenney & Munce, 2003 [16]; Rogeri et al, 2021 [129]; Covinsky et al, 2003 [130]
Co-morbidity management during weather extremes	1. Have a high suspicion for respiratory infections, hypoglycemia, and blood pressure fluctuations during the winter months for patients with co-morbid diabetes, hypertension, and COPD. 2. Encourage seasonal adaptations, including appropriate clothing, effective home heating and avoiding prolonged exposure to the outdoors during temperature extremes.	Temperature extremes exacerbate chronic disease burden and increase mortality risk in elderly patients with hypertension, diabetes, COPD, and various malignancies.	Qi et al, 2024 [74]; Song et al, 2021 [76]; Yang et al, 2024 [82]; Liu et al, 2023 [131]
Medication review and polypharmacy	1. Review medication lists for common offending agents including antipsychotics, antipyretics, β-blockers, anxiolytics, antidepressants, and opioids. 2. Use antihyperglycemics with low risk of hypoglycemia. 3. Review for drug interactions.	Antipsychotics, antipyretics, β-blockers, anxiolytics, antidepressants, and opioids are well documented to affect thermoregulatory dysfunction. Antihyperglycemics can cause hypoglycemia which often manifests as hypothermia.	Sessler, 2009 [106]; Okada et al, 2024 [107]; Fitzgerald, 1980 [108]; Sund-Levander & Grodzinsky, 2009 [53]; Van Marum et al., 2007 [29]
Cognitive and autonomic screening	1. Evaluate patients with persistent low body temperature for autonomic dysfunction or early cognitive decline. 2. Use validated screening tools to assess for cognitive impairment. 3. Refer to geriatrics if there is concern for neurocognitive disorder.	Low body temperature may be a prodromal marker of a major neurocognitive disorder.	Alagiakrishnan et al., 2023 [119]

One of the primary drivers of low body temperature in the elderly is malnutrition and sarcopenia, leading to reduced insulating subcutaneous tissue and faster dissipation of body heat [16, 21]. Sarcopenia also results in reduced muscle mass, leading to lower baseline metabolic rate [129]. Clinicians must ensure that geriatric patients have adequate caloric intake and encourage resistance training to preserve muscle mass. This will serve to safeguard against low body temperature and preserve patients’ independence with activities of daily living as they age [130]. Referrals to a dietician or physiotherapy to assist with these aspects of care are reasonable options as well.

Several common medical conditions in the elderly, including COPD, diabetes, and hypertension have been noted to have poor outcomes in periods of extreme temperatures [74, 76, 82]. Clinicians should have a higher suspicion for respiratory illnesses, hypoglycemia, or exacerbations of COPD during the winter and summer months and with documented low body temperature. Additionally, comorbid elderly patients should limit their exposure to cold snaps, dress appropriately for the weather, and ensure proper heating facilities in their residences [131].

Polypharmacy is likely a major contributor to predisposing to and exacerbating low body temperature in the elderly [99]. Patients may be on several medications that interact with each other and affect thermoregulatory function. Screening for culprit medications such as regular use of antipyretics that blunt the febrile response, antipsychotics which can induce hypothermia, and opting for antihyperglycemics with lower risk of hypoglycemia is prudent in the elderly population [29, 53, 108].

Finally, low body temperatures should not be treated as a benign entity. Low baseline body temperature may be the result of autonomic dysfunction or represent the prodromal phase of a major neurocognitive disorder [119]. In patients with documented low body temperature, we urge clinicians to screen for cognitive impairment.

## Conclusions

Although body temperature is a classic primary vital sign, in the absence of a true fever or hypothermia, it has received little attention from clinicians. Low body temperature may be the result of or predict poor outcomes in elderly patients with several common co-morbidities or geriatric syndromes.

Moving forward, we urge clinicians in the outpatient setting to collect body temperature in all routine follow-up appointments with their geriatric patients. Individuals with documented low body temperature need to be followed more closely for cognitive decline, or conditions that predispose patients to autonomic neuropathy, such as diabetes or COPD. Physicians can mitigate this risk with several practical strategies, including cognitive screening, appropriate management of co-morbidities such as hypertension and diabetes, and addressing polypharmacy to reduce medication-induced reductions in core body temperature. In scenarios with worrisome features of a major neurocognitive disorder, an early referral to geriatrics is not unreasonable.

As the research around low body temperature in older adults is sparse, most of the evidence currently comes from observational data. To clarify whether low body temperature represents a causal risk factor for poor outcomes in geriatric co-morbidities, prospective studies need to be conducted. Current evidence suggests that low body temperature is at minimum a marker of co-morbidity in older adults. While animal studies have shown improved cognition with elevated temperatures in mice models of Alzheimer’s disease, no human clinical trials have demonstrated improved outcomes in the geriatric population through increasing body temperature. Future interventional studies are needed to determine whether correction of low body temperature—through nutritional optimization, medication adjustments, or environmental modifications—can improve clinical outcomes in elderly patients.

## Data Availability

The authors declare that data supporting the findings of this study are available within the article.
